# Megalocytiviruses

**DOI:** 10.3390/v4040521

**Published:** 2012-04-10

**Authors:** Jun Kurita, Kazuhiro Nakajima

**Affiliations:** 1 National Research Institute of Aquaculture, Fisheries Research Agency, Minamiise 519-0423, Japan; 2 Japan Sea National Research Institute, Fisheries Research Agency, Niigata 951-8121, Japan; Email: kazuhiro@affrc.go.jp

**Keywords:** *Iridoviridae*, *Megalocytivirus*, RSIV, ISKNV, TRBIV

## Abstract

The genus *Megalocytivirus*, represented by red sea bream iridovirus (RSIV), the first identified and one of the best characterized megalocytiviruses, Infectious spleen and kidney necrosis virus (ISKNV), the type species of the genus, and numerous other isolates, is the newest genus within the family *Iridoviridae*. Viruses within this genus are causative agents of severe disease accompanied by high mortality in multiple species of marine and freshwater fish. To date outbreaks of megalocytivirus-induced disease have occurred primarily in south-east Asia and Japan, but infections have been detected in Australia and North America following the importation of infected ornamental fish. The first outbreak of megalocytiviral disease was recorded in cultured red sea bream (*Pagrus major*) in Japan in 1990 and was designated red sea bream iridovirus disease (RSIVD). Following infection fish became lethargic and exhibited severe anemia, petechiae of the gills, and enlargement of the spleen. Although RSIV was identified as an iridovirus, sequence analyses of RSIV genes revealed that the virus did not belong to any of the four known genera within the family *Iridoviridae*. Thus a new, fifth genus was established and designated *Megalocytivirus* to reflect the characteristic presence of enlarged basophilic cells within infected organs. Indirect immunofluorescence tests employing recently generated monoclonal antibodies and PCR assays are currently used in the rapid diagnosis of RSIVD. For disease control, a formalin-killed vaccine was developed and is now commercially available in Japan for several fish species. Following the identification of RSIV, markedly similar viruses such as infectious spleen and kidney necrosis virus (ISKNV), dwarf gourami iridovirus (DGIV), turbot reddish body iridovirus (TRBIV), Taiwan grouper iridovirus (TGIV), and rock bream iridovirus (RBIV) were isolated in East and Southeast Asia. Phylogenetic analyses of the major capsid protein (MCP) and ATPase genes indicated that although these viruses shared considerable sequence identity, they could be divided into three tentative species, represented by RSIV, ISKNV and TRBIV, respectively. Whole genome analyses have been reported for several of these viruses. Sequence analysis detected a characteristic difference in the genetic composition of megalocytiviruses and other members of the family in reference to the large and small subunits of ribonucleotide reductase (RR-1, RR‑2). Megalocytiviruses contain only the RR-2 gene, which is of eukaryotic origin; whereas the other genera encode both the RR-1 and RR-2 genes which are thought to originate from *Rickettsia*-like α-proteobacteria.

## 1. Introduction

The family *Iridoviridae* is a family of large dsDNA viruses that display icosahedral symmetry and range in size from 120–200 nm in diameter. The family consists of five genera, *Iridovirus*, *Chloriridovirus*, *Ranavirus*, *Lymphocystivirus* and *Megalocytivirus* [1]. *Megalocytivirus* is the newest genus within the family and, along with the *Ranavirus* and *Lymphocystivirus* genera, contains members that infect cold-blooded vertebrates. Although not yet formally adopted by the International Committee on the Taxonomy of Viruses (ICTV), the subfamily designation *Chordiridovirinae* has been proposed by Chinchar *et al*. for this group [2]. Red sea bream iridovirus (RSIV) is the first reported member of this genus and is the causative agent of severe disease in mainly East and Southeast Asian maricultured fish species. In recent years, many closely related viruses have been reported from this area. In this report, the history of research on RSIV is reviewed and the relationship between RSIV and other members of the genus *Megalocytivirus* is discussed. 

## 2. First Report of Megalocytiviral Disease: Clinical Signs, Pathology and Epidemiology of the Red Sea Bream Iridovirus Disease (RSIVD)

**Figure 1:**
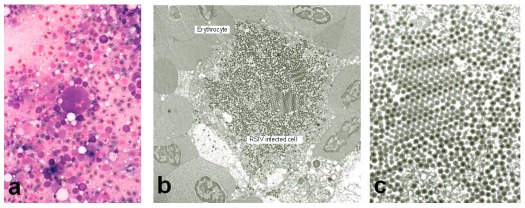
(**a**) Giemsa-stained impression smears of the spleen of RSIV-infected red sea bream display enlarged cells characterized by basophilic staining. (**b**) Electron micrograph of RSIV‑infected spleen cells. (**c**) Higher magnification of the virions seen in panel B. All photographs were kindly provided by Dr. K. Inouye.

The first outbreak of megalocytivirus-induced disease was recorded in cultured red sea bream (*Pagrus major*) in Japan in 1990 and designated red sea bream iridovirus disease (RSIVD) by Inouye *et al.* [3]. Since 1991, the disease has caused mass mortalities in more than thirty species of cultured marine fish in the western part of Japan. The disease infects mainly fingerlings but market‑sized fish are also affected. The range of susceptible hosts consists mainly of species within the order Perciformes, but some species belonging to the orders Pleuronectiformes and Tetraodontiformes are also affected [4,5]. In the case of RSIVD in Japan, the disease occurs mainly in the summer, a period of relatively high water temperature. Diseased fish are lethargic, swim helplessly, and show severe anemia, petechiae of the gills, and enlargement of the spleen with 20–60% mortality. Histopathology is characterized by development of enlarged cells in the spleen, heart, kidney, liver, and gills (Figure 1a) that display basophilic characteristics when stained with Giemsa [3]. These enlarged cells have been termed inclusion body-bearing cells and their appearance is pathognomonic for RSIVD [6,7].

## 3. Virological Studies and Pathogenicity of the Agent

Icosahedral virions are found within the cytoplasm of enlarged cells (Figure 1). Each virion consists of a central electron-dense core (120 nm), an electron translucent zone, and measures 120–200 nm in diameter. Feulgen staining of enlarged cells demonstrated the presence of DNA in the viral inclusions. These morphological features suggested that the virus belonged to the family *Iridoviridae* and the virus was named red sea bream iridovirus (RSIV) after the species from which it was first isolated. RSIV replicated slowly and produced cytopathic effect (enlarged and rounded cells) in cultures of RTG-2, CHSE-214, FHM, BF-2 and KRE-3 cells at 20–25 °C. However, RTG-2, CHSE‑214, and FHM cultures were not suitable for diagnosis because CPE developed very slowly and resulting viral titers (an indication of susceptibility) were low. Intraperitoneal inoculation into red sea bream fingerlings of a cell-free preparation that was prepared from the spleens of infected fish and filtered by passage through a 0.45 micron membrane induced pathological changes similar to those observed in naturally diseased fish [3]. Subsequently, the biological and physico-chemical properties of this virus were studied [8]. It was shown that the virus replicated in BF-2 and KRE-3 cells at an optimal temperature of 25 °C but that serial passage of the virus in both BF-2 and KRE-3 cells resulted in a gradual decrease in infectivity and loss of infectious virus. Consistent with the presence of a lipid membrane, both chloroform and ether treatment destroyed the infectivity of RSIV. Furthermore, the virus was acid (pH 3.0) and heat (56 °C 30 min) labile, and iododeoxyurdine inhibited viral replication by 3 log units. Membrane filtration suggested that virion diameter was less than 220 nm. The pathogenicity of RSIV isolates from various cultured marine fish species was confirmed by experimental infection using red sea bream as the host. In addition, pathogenicity of RSIV isolated from red sea bream for Japanese amberjack (*Seriola quinqueradiata*) was also demonstrated [9]. These results showed RSIV to be a newly identified and unique piscine iridovirus which, although initially isolated from red sea bream, displayed a remarkably broad host range. 

## 4. Establishment of a New Genus within the Family *Iridoviridae*

**Figure 2:**
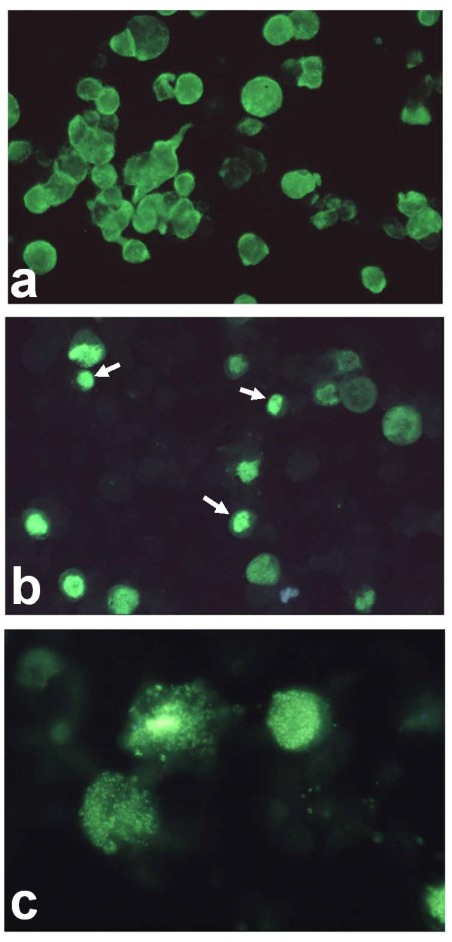
Immunofluorescent assay. (**a**) RSIV-infected Grunt Fin(GF) cells were incubated with monoclonal antibody M10 and ballooned, infected cells were identified by the presence of diffuse staining throughout the cell; (**b**) RSIV-infected GF cells were incubated with polyclonal rabbit anti-RSIV serum which detects structural proteins. In contrast to panel A, staining is seen only within viral assembly sites. Viral assembly sites are indicated by arrows.The assembly site becomes granulated and is gradually dispersed throughout the whole cell (Panel c);(**c**) Higher magnification of stained cells filled with granules.

Using a unique culture method involving persistently-infected Grunt Fin (GF) cells and subsequent virus purification, both polyclonal and monoclonal antibody preparations [10] specific to RSIV were produced. Comparison of infected cells stained by monoclonal antibody M10, which likely recognizes a virus induced non-structural protein which is expressed prior to the major capsid protein, and rabbit anti-RSIV polyclonal antibody, which recognizes viral structural proteins, showed (Figure 2) that the M10 target is expressed in all infected cells and widely distributed throughout the cell (Panel a), whereas structural proteins (Panel b) are expressed in only a fraction of infected cells and confined primarily to viral assembly sites. The observation that most infected cells failed to express viral structural protein explains why it is difficult to culture virus. Further serological analysis showed that RSIV was quite different from previously characterized fish and amphibian ranaviruses, *i.e.*, no cross was noted between megalocytiviruses and frog virus 3 (FV3), epizootic haematopoietic necrosis virus (EHNV), European sheatfish virus (ESV) and Singapore grouper iridovirus (SGIV) [10,11]. In addition, partial genomic analysis was performed and the sequences of the DNA polymerase and ATPase genes were identified and deposited with Genbank (Acc. Nos. AB007366 and AB007376, respectively) [12]. Analysis of those sequences clearly showed that RSIV did not belong to the genera *Lymphocystiviru*s, *Ranavirus* or *Iridovirus*, but rather represented a fifth genus equidistant, phylogenetically, from other genera within the family *Iridoviridae*. Furthermore, the RSIV major capsid protein (MCP) gene, which has served as a target for phylogenetic analyses, was also identified (Acc. AB080362) after the complete genomic sequence of a closely related virus, infectious spleen and kidney necrosis virus (ISKNV), was reported [13]. Reflecting the presence of characteristically enlarged cells within the spleen and other organs of infected fish, the new genus was named *Megalocytivirus*.

## 5. Diagnostic Methods for RSIVD

Analysis of virus-infected cells using Giemsa-stained impression smears of the spleen (Figure 1a) [3] has been commonly used for the rapid diagnosis of RSIV-infected fish. However, this method provides only a presumptive diagnosis and does not confirm the presence of RSIV. Moreover, isolation of virus in cell culture using susceptible cell lines such as BF-2, KRE-3 and GF is time‑consuming and may require 1 to 2 weeks to complete. Therefore, M10, a monoclonal antibody that recognizes a 180–230 kDa virus-induced, non-structural protein was selected and used for development of a rapid test for RSIV detection [14]. This assay can be used, not only with spleen impression smears from infected fish, but also with infected cell cultures and tissue sections. The use of monoclonal antibody M10 is described in the Manual of Diagnostic Methods for Aquatic Animals published by the World Organization for Animal Health (OIE) and is applicable to both RSIV and ISKNV. Other rapid and more sensitive confirmatory diagnostic methods, such as PCR [12,15,16,17,18], have also been developed. Unfortunately, one of the PCR tests [17] was subsequently shown to be incorrect since it detected a contaminant bacterial species closely related to *Acholeplasma laidlawii* rather than RSIV.

## 6. Control of RSIVD by Vaccination

For the control of RSIVD, an effective formalin-inactivated vaccine was developed by Nakajima *et al*. [19,20]. This injectable vaccine is now commercially available in Japan for red sea bream as well as for fish belonging to the genus *Seriola*, striped jack (*Pseudocaranx dentex*), Malabar grouper (*Epinephelus malabaricus*) and orange-spotted grouper (*Epinephelus coioides*). Protection of fish species belonging to the genus *Oplegnathus* by vaccination is difficult because of the high susceptibility of these species to RSIV infection. This vaccine represents the first viral vaccine for marine fish in the world. A vaccine has also been developed in Taiwan for Taiwan grouper iridoviral disease, a similar megalocytiviral disease.

## 7. Diseases Similar to RSIVD in East and Southeast Asia

During the past 15 years, RSIV-like viruses from cultured marine and freshwater fish and tropical ornamental fish have been reported mainly in East and South East Asia [2,6,7,21,22,23,24,25,26,27,28,29,30,31,32,33,34,35,36,37]. The various isolates include infectious spleen and kidney necrosis virus (ISKNV) (22), Taiwan grouper iridovirus (TGIV) [2,22], turbot reddish body iridovirus (TRBIV) [28], rock bream iridovirus (RBIV) [30] and dwarf gourami iridovirus [6,33]. These viruses are closely related to RSIV morphologically and genetically, and the pathology of infected fish is similar to RSIVD. Given the wide host range of RSIV, some of these viruses are likely isolates of RSIV, whereas others likely represent different, but closely related, viral species. Genetic analysis suggests that two additional viral species exist in the genus *Megalocytivirus. *One of those is represented by ISKNV isolated from freshwater Chinese perch (*Siniperca chuatsi*) and another by TRBIV isolated from turbot (*Scophthalmus maximus*). 

## 8. Complete Genomic Analysis of Megalocytiviruses

**Figure 3:**
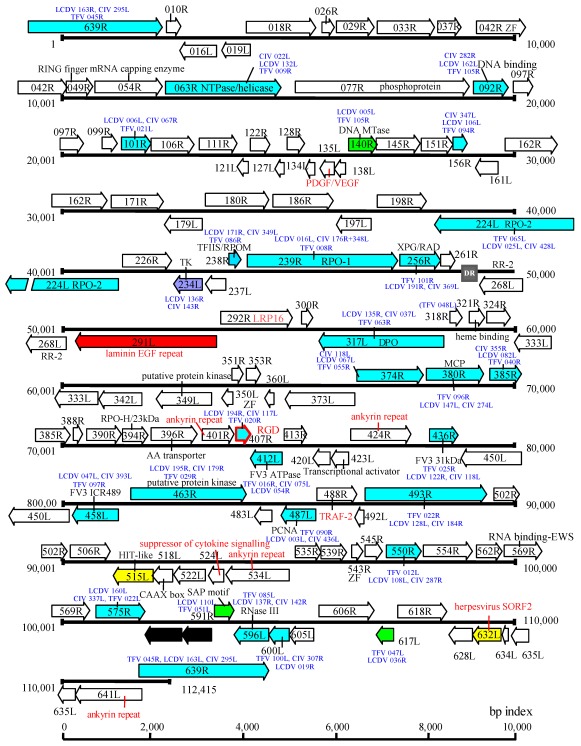
Genome structure of RSIV-Ehime 1 strain. Blue arrows indicate genes common among the *Megalocytivirus*, *Ranavirus*, *Lymphocystivirus* and *Iridovirus *genera. Green arrows show genes found among all vertebrate iridoviruses. The single purple arrow shows a gene which is not encoded by the genus *Ranavirus. *Yellow arrows show RSIV‑specific genes, red annotations indicate putative virus-host interaction genes, and the red arrow shows a possible attachment protein gene.

**Figure 4:**
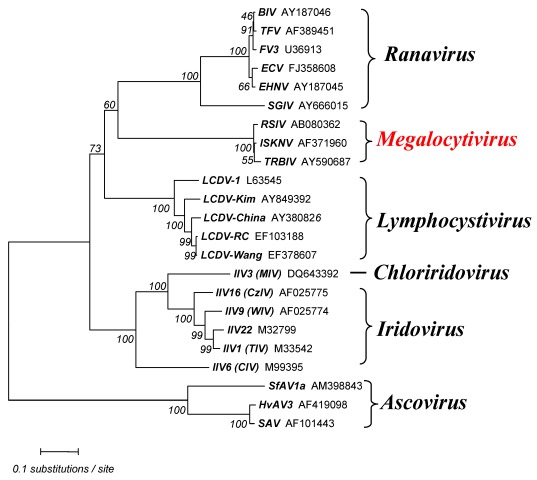
Iridovirus phylogeny. The tree was constructed based on amino acid sequences of the major capsid protein (MCP) by the neighbor-joining method [43] using MEGA (version 4) [44]. The Poisson correction was selected as the distance parameter and pair‑wise deletion was used to handle sequence gaps. The numbers indicate the percentage bootstrap support for each node following 1000 replicates. Ascoviral homologs were added as an out-group. Accession numbers are attached to each operational taxonomic unit (OTU).

The megalocytiviral genome is a linear, dsDNA molecule that is circularly permuted and terminally redundant. However, at this time, the percent terminal redundancy is not known. Currently, complete genomic sequence information is known for five megalocytiviruses, ISKNV (AF371960) [13], RSIV‑Ehime1 strain (BD143114, AB104413) [29,38] rock bream iridovirus (RBIV) (AY532606) [39], orange-spotted grouper iridovirus (OSGIV) (AY894343) [40], TRBIV (GQ273492) [41], and large yellow croaker iridovirus (LYCIV) (AY779031). GC content and genome lengths range from 53–55% and 110,104–112,636 bp, respectively, and are more similar to ranaviruses than to viruses within other iridoviral genera. Similar to other vertebrate iridoviruses, it is likely that megalocytivirus genomes are highly methylated, as evidenced by the existence of a DNA methyltransferase (DMT) gene and the difficulty of cloning of viral genomic fragments using *E. coli* strains which lack tolerance for methylated DNA. There are 116 open reading frames (ORFs) encoded by RSIV Ehime-1 (Figure 3) and this number is similar to that seen in other megalocytiviruses. In the case of RSIV, most ORFs are non-overlapping and all are intronless. RSIV genes are divided into two categories: (1) those genes common to all iridoviruses (~30 genes); and (2) RSIV-specific genes (86 genes). The former includes genes encoding the major capsid protein (MCP), the viral DNA polymerase, the two largest subunits of the viral RNA polymerase, an XPG/RAD2 family gene, and DMT (common to only vertebrate iridoviruses). Supporting the suggestion that RSIV belongs to a fifth genus within the family *Iridoviridae*, phylogenetic analyses of the MCP indicates that megalocytiviruses cluster closely together and form a distinct group apart from those containing ranaviruses or lymphocystiviruses (Figure 4).

Analyses of various megalocytivirus genomes provided important information regarding the evolution of the family. Among iridoviruses, major replicative and transcriptive enzymes likely originated from their eukaryotic hosts [29,42]. The presence of similar genes among all genera within the family suggests that the ancestral iridovirus must have accessed genes from its eukaryotic host prior to the later differentiation of the family into the current five genera [29]. Genes originating from eukaryotes are also commonly found in other large DNA virus families, but iridoviral genes of eukaryotic origin display higher levels of identity to eukaryotic genes than those in other families [29]. It is thought that the evolution of iridovirus genes takes place more slowly than that seen in other families of large DNA viruses. This may be explained by the acquisition by the ancestral iridovirus of a XPG/RAD family gene which functions in DNA repair and helps maintain the integrity of the genome [29].

**Figure 5:**
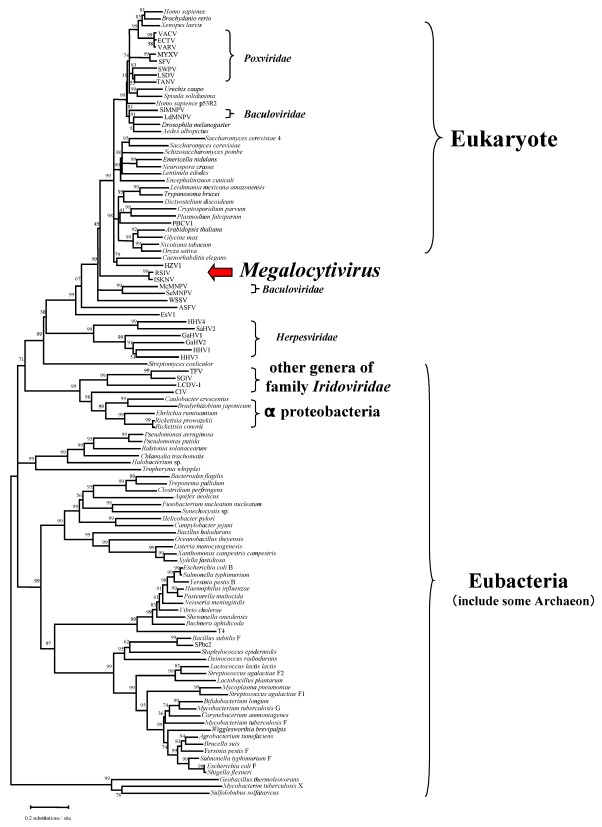
Phylogenetic analysis of the small subunit of ribonucleotide reductase (RR-2). The tree was constructed by the neighbor-joining method [43] using MEGA (version 4) [44]. Poisson correction was selected as the distance parameter and pair-wise deletion was chosen to handle gaps. The numbers indicate the percentage bootstrap support for each node from 1,000 replicates.

Iridovirus homologs of ribonucleotide reductase, an enzyme that plays a critical role in eukaryotic DNA synthesis, deserves special mention. Genes encoding the large and small subunits of ribonucleotide reductase (RR-1 and RR-2, respectively) are found in all iridoviruses except the genus *Megalocytivirus* and are thought to be derived from *Rickettsia*-like eubacteria (Figure 5). Moreover, the RR-1 gene from the genus *Iridovirus* has an intein structure, whereas those of *Ranavirus* and *Lymphocystivirus*, the vertebrate iridoviral group, do not. RR-1 with an included intein is very rare and is only seen in particular bacteria and phage. In contrast, megalocytiviruses encode only the RR-2 gene and it shows only very low homology to those of other iridoviruses. Phylogenetic analysis suggests that the megalocytivirus RR-2 gene appears to have originated from a past eukaryotic host. Thus, the absence of a virally-encoded RR1 gene, as also seen among some poxviruses, suggests that formation of a functional RR tetramer likely requires the association of the megalocytivirus RR2 with host RR1 [45]. Phylogenetic analysis of RR genes suggests that megalocytivirus RR genes are evolutionarily divergent from those of other iridovirus genera (Figure 4). Other megalocytivirus genes of interest include a cytokine suppressor gene and three ankyrin repeat-containing genes that possibly encode repressors of the host immune response. In addition, a laminin EGF-repeat-containing gene product or a putative cell attachment protein containing the RGD motif may serve as viral receptors. 

## 9. Phylogenetic Analysis and Host Range of Megalocytiviruses

**Figure 6:**
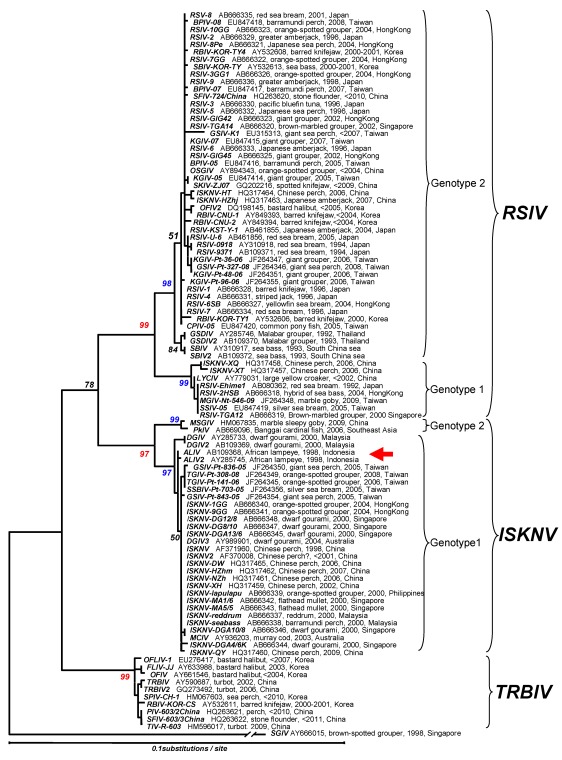
Phylogenetic analysis of the megalocytivirus MCP. The tree was constructed by the neighbor-joining method [43] using MEGA (version 4) [44]. Tamura and Nei’s parameters and pair-wise deletion of gap handling were selected. The numbers indicate the percentage bootstrap support for each node from 1,000 replicates. The tree was rooted using MCP sequences from Singapore grouper iridovirus (SGIV), genus *Ranavirus*. Stop codons were excluded from the analysis.The red arrow shows the position of African lampeye iridovirus (ALIV). Accession numbers, host, year sampled, and location of isolation are attached to each OTU.

**Figure 7:**
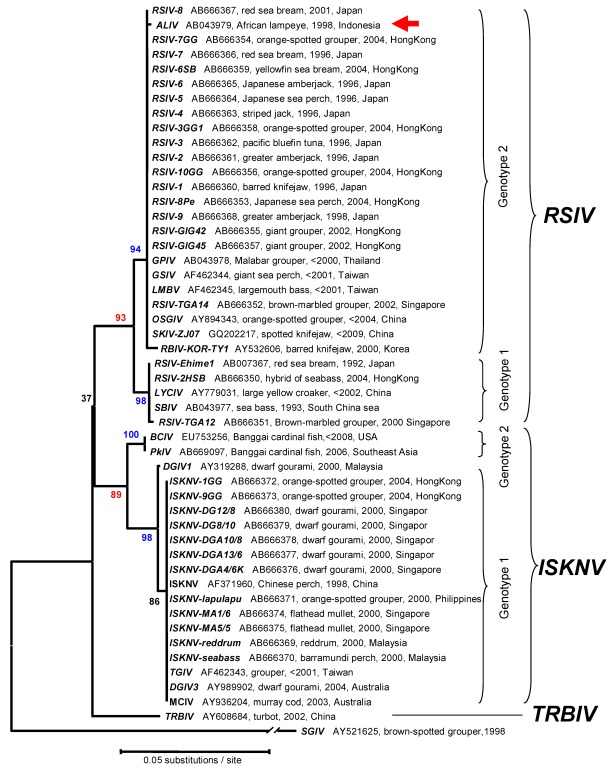
Phylogenetic analysis. This tree, based on the megalocytivirus ATPase gene, was constructed as described in the legend to Figure 6. Accession numbers, host, year sampled, and locations are attached to each OTU.

Phylogenetic analyses using MCP and ATPase genes have been performed for multiple megalocytivirus isolates. Analysis of the MCP (Figure 6) and ATPase (Figure 7) genes show that the genus *Megalocytivirus* can be divided into three clusters represented by RSIV, ISKNV and TRBIV [29,30,35,36,37]. Although the amino acid divergence among these three clusters is small, it is about the same magnitude as that seen among BIV, TFV, and FV3 (genus *Ranavirus*, Figure 4) suggesting that RSIV, ISKNV, and TRBIV could be considered distinct viral species. When we focused on the RSIV cluster, the RSIV-type viruses can be further divided into two subclusters: genotype I, to which RSIV Ehime-1 (the type strain of RSIV) belongs, and genotype II, which includes the other major RSIV strains found in Japan. It is clear that the virus from Thailand designated grouper sleeping disease virus (GSDIV) [24], RBIV isolated in South Korea, and other viruses found in East and Southeast Asia are likely strains of the same viral species and belong to RSIV genotype II. Although GSDIV was reported as the causative agent of grouper sleeping disease [24], Singapore grouper iridovirus (SGIV), a member of the genus *Ranavirus*, was also reported to be the causative agent of the disease [46]. The host range for RSIV is very wide and includes most of the important maricultured fish species. The fact that viruses similar to Japanese RSIV are also found in Southeast Asia suggests that RSIV was introduced into Japan from Southeast Asia via imported fish seedlings, a hypothesis consistent with the one-way movement of seedlings into Japan.

The second tentative species within the genus Megalocytivirus is represented by ISKNV and related viruses. The ISKNV species comprises a variety of isolates including Taiwan grouper iridovirus (TGIV) [2,20], dwarf gourami iridovirus (DGIV) [23,33], mullet iridovirus (MIV) [27] and others. ISKNV-like viruses show less genetic variation than RSIV and differ in biological characteristics, e.g., marine isolates of ISKNV are easier to propagate in culture than RSIV but DGIV, one of the freshwater strains of ISKNV, is more difficult to propagate in culture than RSIV. Recently, sequences of the MCP and ATPase genes of viruses isolated from Bangaii cardinal fish (*Pterapogon kauderni*) and marble sleepy goby (*Oxyeleotris marmorata*) have been deposited within Genbank (Acc. Nos. HM067835, AB669096, EU753256, AB669097). Phylogenetic analysis revealed that these viruses are also members of ISKNV cluster but differ from all other reported ISKNV isolates and belong to a new genotype (genotype II) (Figure 6). Moreover, the isolation of an ISKNV-like virus in North America from imported Bangaii cardinal fish underscores the need to ensure that infected fish are not introduced into megalocytivirus-free regions. Clearly, the host range of ISKNV is relatively broad but freshwater and brackish water fish species are the predominant species affected. African lampeye iridovirus (ALIV) is considered to be the same viral species as ISKNV based on MCP gene sequence analysis, but it clusters with RSIV by ATPase gene sequence analysis (Figure 7). It is not clear whether the origins of these two entries of ALIV sequences are the same or not, even though the sequence data were deposited by the same researcher [24]. There are two possibilities for this result: either viruses similar to ISKNV and RSIV were both isolated from African lampeyes or ALIV is a chimeric virus displaying the MCP of ISKNV and the ATPase of RSIV. Further research is required to resolve this question. Recently, a number of studies have been undertaken to identify gene function in ISKNV [47,48,49,50,51]. These expanding biochemical studies will clarify the function of specific ISKNV genes and indicate whether megalocytiviruses display replicative strategies unique to this viral genus. 

TRBIV, the last tentative species within this genus, includes flounder iridovirus (FLIV-JJ) [31] and other viruses from bastard halibut (*Paralichthys olivaceus*). Interestingly, viral hosts belong mainly to the order Heterosomata, (see Figure 6). Exceptions include barred knifejaw (*Oplegnathus fasciatus*), the host for RBIV-KOR-CS [30], a strain of TRBIV, and sea perch (*Lateolabrax* sp.). In the latter case, sequence data from a virus designated perch iridovirus CH-1 (Acc. HM067603) indicated that the isolate was related to TRBIV. Barred knifejaw and the related spotted knifejaw (*O. punctatus*) are known to be markedly susceptible to RSIV (and TRBIV) and vaccination of these species is not effective in protecting them from RSIVD. Given the impact of megalocytiviruses on mariculture, further research is needed to determine whether TRBIV should be considered, along with RSIV and ISKNV, as an OIE-reportable disease. 

## 10. Distribution of Megalocytiviruses

Although genotype II predominates, both RSIV genotypes I and II are widely distributed in East and Southeast Asia. In contrast, in Japan and South Korea outbreaks, with the exception of a single outbreak caused by genotype I virus in Ehime prefecture Japan in 1992, have been caused by genotype II. Until recently, natural hosts of RSIV were thought to be restricted to marine fish. Recently, genotype and host range analysis of megalocytiviruses infecting Chinese perch, the first reported host of ISKNV, were undertaken in China [36]. The result showed that not only ISKNV, but also RSIV genotype I (named to ISKNV-XQ, and -XT,) and genotype II (named to ISKNV-HT and -Hzhj) were present. These are first reports of RSIV from freshwater fish. Based on these findings, it is possible that this freshwater fish species may be a “mixing vessel” for megalocytiviruses and underlies the danger of identifying a virus based only on the species of fish infected. Except for ornamental fish, the distribution of ISKNV remains restricted to Southeast Asia, Taiwan and China and has not yet been found in Japan or South Korea. In contrast to RSIV and ISKNV, TRBIV has only been reported from China and South Korea. The distribution of this virus is still restricted to areas around the Yellow Sea in East Asia and has not yet been found in Southeast Asia. Hong Kong, a base for fish seedling export, is a hotspot for megalocytiviruses. Presence of ISKNV and RSIV genotypes I and II has been confirmed in Hong Kong and it is possible that all Asian megalocytiviruses, except TRBIV, spread from this area.

## 11. Differential Diagnostic PCR Primers for Megalocytiviral Diseases

Except for diseases found in freshwater ornamental fish, the OIE Reference Laboratory for RSIVD designates both genotypes of RSIV and ISKNV as causative agents of RSIVD. The reason for including ISKNV is the severe impact of this virus on many kinds of maricultured fish species in China, Taiwan and many Southeast Asian countries. Megalocytiviruses of freshwater ornamental fish, such as DGIV and ALIV, have almost no opportunity to affect marine fish culture even if these viruses are pathogenic to marine fish species. TRBIV disease is not yet listed in the OIE manual as a reportable infection because the virus is considered to be an agent of low pathogenicity, but this suggestion needs to be more thoroughly tested. Thus, it is important that a simple diagnostic test be developed that is able to differentiate among these viruses. In addition, the presence of multiple viral strains has caused problems in designing primers for diagnostic PCR. Using genomic DNA sequence information for the MCP gene, it is possible to propose new PCR primers for differentiation among the three viral species of the genus *Megalocytivirus* viz. RSIV, ISKNV and TRBIV (Table 1). The forward primer MCP-uni332-F3 (5'-aggtgtcggtgtcattaacgacctg-3') and the reverse primer MCP-uni1108-R8 (5'-tctcaggcatgctgggcgcaaag-3') amplifies a fragment 777bp in length and are proposed as a universal PCR primer pair for all megalocytiviruses. Forward primer MCP-excT37-F1 (5'-ttcatcgacatctccgcgttt-3') and reverse primer MCP-excT512-R1 (5'-aatgggcaaattaaggtagrcg-3′) pair, which amplify a fragment 486bp in length are proposed as the RSIV- and ISKNV-specific primer pair, although the lack of reactivity to TRBIV needs to be confirmed. In addition, as a TRBIV-specific primer pair, MCP-specT37-F1 (5'-ttcatcgacatctccgctttc-3') and MCP-specT490-R1 (5'-tstgaccgttggtgataccggag-3') pair, which amplify a fragment 453bp in length is proposed. While it is hoped that these primers have the desired specificities, they are only tentative until testing with further TRBIV samples is undertaken. For the purpose of differentiating RSIV and ISKNV, the following primer sets are used. Primer set MCP-specR697-F4 (5'-cccgcactgaccaacgtgtcc-3') and MCP-specR888-R6 (5'-cacagggtgactgaacctcaggtcg-3') which amplifies a fragment 191bp in length is specific for RSIV. Primer set MCP-specI465F3 (5'-ggtggccggcatcaccaacggc-3') and MCP-specI879-R6 (5'-cacggggtgactgaacctg-3') which amplifies a fragment 415bp in length are specific for ISKNV. Please note that the specificity of primer sets for RSIV and ISKNV has been confirmed but the specificity of the TRBIV primer sets remains unknown. The OIE Reference Laboratory for RSIVD welcomes comments with regard to information on the proposed primers for improvement of diagnostic PCR tests for RSIVD.

**Table 1:** Primer pairs for universal and selective amplification of megalocytivirus isolates.

Viruses Identified	Forward Primer ^a^	Reverse Primer	Amplicon Size (bp)
RSIV, ISKNV, TRBIV	MCP-uni332-F3	MCP-uni1108-R8	777
RSIV, ISKNV	MCP-excT37-F1	MCP-excT512-R1	486
TRBIV	MCP-specT37-F1	MCP-specT440-R1	453
RSIV	MCP-specR674-F4	MCP-specR888-R6	191
ISKNV	MCP-specI465-F3	MCP-specI879-R3	415

^a^ The sequence of each primer is listed in the preceding paragraph.
